# Readiness of Dental Professionals to Adopt Artificial Intelligence for Cone-Beam Computed Tomography Interpretation: A Cross-Sectional Survey

**DOI:** 10.7759/cureus.115237

**Published:** 2026-08-26

**Authors:** Sameer Chauhan, Arani Roy, Suraiya Khan, Drishti Palwankar, Rinku N Adwani, Karan Singh

**Affiliations:** 1 Department of Prosthodontics, K. M. Shah Dental College and Hospital, Sumandeep Vidyapeeth (Deemed to be University), Vadodara, IND; 2 Department of Dentistry, Balurghat District Hospital, Balurghat, IND; 3 Department of Public Health, Johns Hopkins Bloomberg School of Public Health, Baltimore, USA; 4 Department of Conservative Dentistry and Endodontics, Faculty of Dental Sciences, Shree Guru Gobind Singh Tricentenary University, Gurugram, IND; 5 Department of Orthodontics, Vidarbha Youth Welfare Society (VVWS) Dental College and Hospital, Amravati, IND; 6 Department of Orthodontics, Rayat Bahra Dental College and Hospital, Mohali, IND

**Keywords:** artificial intelligence, cone-beam computed tomography, cross-sectional survey, dental professionals, readiness

## Abstract

Introduction: Artificial intelligence (AI) is being increasingly incorporated into cone-beam computed tomography (CBCT) interpretation to support diagnostic accuracy and clinical decision-making. However, successful implementation depends on the readiness and acceptance of the dental professionals. This study aimed to evaluate the readiness of dental professionals to adopt AI for CBCT interpretation and identify the demographic and professional factors associated with AI readiness.

Materials and methods: A cross-sectional questionnaire-based survey was conducted among dental professionals using a structured questionnaire for demographic information (section 1) and AI readiness assessment (section 2). The questionnaire was developed by a multidisciplinary panel of dental specialists, and pilot-tested and assessed for internal consistency before administration. Responses were collected electronically on a five-point Likert scale. Descriptive statistics were used to summarize the participant characteristics and readiness scores. Internal consistency was evaluated using Cronbach's alpha. Independent t-tests, Spearman's rank-order correlation, and multiple linear regression analyses were performed to identify the factors associated with AI readiness.

Results: A total of 380 complete responses were included in the analysis. The questionnaire demonstrated good internal consistency (Cronbach's α = 0.87). The largest proportion of participants belonged to the age group of 31-40 years, followed by the age group of 20-30 years; 209 (55.0%) participants were male. Most respondents were Master in Dental Surgery (MDS) graduates, and 114 (30.0%) had 5-10 years of clinical experience. Younger participants (≤40 years), qualified postgraduate professionals, those with <10 years of clinical experience, and academic practitioners demonstrated significantly higher AI readiness scores than their counterparts (p < 0.05). Correlation analysis showed that age (r = −0.34, p < 0.001) and clinical experience (r = −0.32, p < 0.001) were negatively associated with AI readiness, whereas qualification demonstrated a positive correlation (r = 0.28, p < 0.001). Multiple linear regression revealed that greater clinical experience independently predicted lower readiness (β = −0.28, p < 0.001), whereas higher qualification (β = 0.15, p = 0.002), academic practice setting (β = 0.10, p = 0.042), and prior AI training (β = 0.17, p = 0.001) were significant positive predictors. The regression model explained 25% of the variance in AI readiness (R² = 0.25; adjusted R² = 0.24; p < 0.001).

Conclusion: Dental professionals demonstrated an encouraging level of readiness to adopt AI for CBCT interpretation, although acceptance varied according to demographic and professional characteristics. Educational qualification, professional experience, and prior AI exposure were seen to be important determinants of readiness. Incorporating structured AI education and continuing professional training into dental curricula and clinical practice may facilitate the integration of AI-assisted CBCT interpretations, improve diagnostic efficiency, and support evidence-based patient care.

## Introduction

Artificial intelligence (AI) has emerged as one of the most transformative technologies in healthcare, offering innovative solutions to improve diagnostic accuracy, efficiency, and clinical decision-making [1]. In dentistry, AI applications have expanded rapidly across multiple specialties, including oral radiology, orthodontics, prosthodontics, endodontics, and oral pathology [2]. Among various imaging modalities, cone-beam computed tomography (CBCT) has become an indispensable diagnostic tool because of its ability to provide three-dimensional (3D) visualization of craniofacial structures with high spatial resolution. CBCT is routinely used for implant planning, impacted tooth assessment, temporomandibular joint evaluation, endodontic diagnosis, airway analysis, and maxillofacial [3,4]. However, interpreting CBCT scans requires considerable expertise because the large volume of image data increases the possibility of overlooking clinically significant findings.

Recent advances in deep learning and machine learning have enabled AI systems to detect anatomical landmarks, plan for implants, identify pathological lesions, segment anatomical structures, and assist clinicians in generating diagnostic reports from CBCT images [5,6]. These technologies have the potential to reduce diagnostic errors, improve consistency, shorten interpretation times, and enhance workflow efficiency. Rather than replacing clinicians, AI is increasingly viewed as a decision-support tool that complements professional judgment and facilitates evidence-based patient care [7].

Despite these technological advancements, successful implementation of AI in routine dental practice depends largely on the readiness and acceptance of dental professionals. Factors such as knowledge of AI, perceived usefulness, trust in AI-generated interpretations, previous training, clinical experience, and concerns regarding professional autonomy may significantly influence adoption [8]. Understanding these perceptions is essential for designing educational programs, developing user-friendly AI systems, and promoting the integration of AI into clinical practices. Although several studies have evaluated AI performance in dental imaging, there is limited evidence regarding the preparedness of dental professionals to incorporate AI-assisted CBCT interpretation into everyday practice [9,10].

Therefore, this cross-sectional survey was conducted to evaluate the readiness of dental professionals to adopt AI-assisted CBCT interpretation. The specific objectives were to: (i) assess self-reported AI readiness, including knowledge, attitudes, confidence, and willingness to adopt AI-assisted CBCT interpretation, (ii) compare AI readiness scores according to demographic and professional characteristics, including age, sex, educational qualification, clinical experience, and practice setting, and (iii) identify demographic and professional factors independently associated with AI readiness using multiple linear regression analysis.

## Materials and methods

Study design, setting, and duration

This cross-sectional questionnaire-based survey was conducted at the Department of Prosthodontics and Crown and Bridge, K. M. Shah Dental College and Hospital, Sumandeep Vidyapeeth (Deemed to be a University), Gujarat, India. The study was carried out over a period of six months (January 2025 to June 2025) following approval from the Sumandeep Vidyapeeth Institutional Ethical Committee (reference no: SVIEC/Dent/2024/Dec/205, dated December 24, 2024). This study adhered to the ethical principles of the Declaration of Helsinki. Participation was voluntary and informed consent was obtained from all participants before completing the questionnaire.

Study population and eligibility criteria

The study population comprised registered dental professionals, including undergraduate qualified dentists (Bachelor of Dental Surgery (BDS)), postgraduate specialists (Master in Dental Surgery (MDS)), fellowship or diploma holders, and PhD-qualified dental professionals who were actively involved in clinical practice, teaching, or both. Eligible participants were aged 20 years or older, currently practicing dentistry in India, and able to understand English, the language of the questionnaire. Dental interns, undergraduate students, retired professionals, incomplete questionnaire responses (more than 20% missing data), duplicate submissions, and respondents who declined to consent were excluded from the study.

Sample size estimation

The sample size was calculated using the G*Power software version 3.1.9.7 (Heinrich Heine University Düsseldorf, Düsseldorf, Germany), assuming a 95% confidence level, 5% margin of error, and a conservative expected proportion of 50%, as no previous Indian study has established the prevalence of AI readiness among dental professionals. The minimum sample size required was estimated to be 385 participants. To compensate for incomplete responses and potential non-responses, an additional 10% was added, resulting in a target sample size of 423 participants. A total of 380 complete responses were ultimately received and included in the final statistical analysis, after excluding incomplete and duplicate questionnaires.

Questionnaire development

A structured, self-administered questionnaire was specifically developed for this study, following an extensive review of the literature on AI adoption, technology acceptance in healthcare, and AI applications in dental radiology. The questionnaire was prepared collaboratively by a multidisciplinary panel of specialists comprising experts in Prosthodontics, Orthodontics, Oral Medicine and Radiology, Conservative Dentistry and Endodontics, and Public Health Dentistry. The panel evaluated the relevance, clarity, comprehensiveness, and clinical applicability of each item through multiple rounds of discussions until a consensus was reached. The final questionnaire consisted of 17 items divided into two sections (see Appendices).

The first section included five demographic questions addressing age, sex, highest professional qualification, years of clinical experience, and primary practice setting. Prior AI training was assessed using a single self-reported dichotomous question included in the demographic section of the questionnaire: "Have you received any formal training in AI or AI-assisted dental applications?" Participants selected either "Yes" or "No." Formal AI training was defined as participation in structured educational activities, including workshops, continuing dental education (CDE) programs, certification courses, webinars, or institutional training related to AI applications in dentistry. Responses were coded as Yes = 1 and No = 0 for statistical analysis.

The second section comprised 12 questions assessing readiness to adopt AI for CBCT interpretation, including knowledge of AI, perceived usefulness, confidence in AI-assisted diagnosis, trust in AI-generated interpretations, willingness to incorporate AI into routine practice, perceived educational needs, and concerns regarding replacing professional expertise with AI. One negatively worded statement concerning the perceived reduction in the role of dental professionals (B9) was reverse-scored during the statistical analysis to minimize acquiescence bias. This assessed concerns regarding a potential reduction in the professional role of dental practitioners following AI adoption. Because greater endorsement of this item was initially conceptualized as a potential barrier to AI adoption, it was reverse-scored when calculating the composite readiness score. However, this item may also represent a distinct dimension related to perceived professional threat or autonomy concerns; therefore, its contribution to the overall readiness construct was interpreted cautiously. Each readiness item was scored using a five-point Likert scale (0 = strongly disagree, 1 = disagree, 2 = neutral, 3 = agree, and 4 = strongly agree), yielding a total possible score ranging from 0 to 48, with higher scores indicating greater readiness to adopt AI-assisted CBCT interpretation.

Age and clinical experience were recorded in predefined categories for descriptive and subgroup analyses. For correlation and multivariable regression analyses, age and clinical experience were additionally entered as continuous variables. Educational qualification, sex, practice setting, and prior AI training were treated as categorical variables. Clinical experience was additionally categorized as <10 years and ≥10 years for subgroup comparison, while the continuous measure was retained for correlation and regression analyses. Categorical variables were entered into regression analysis using appropriate indicator (dummy) coding, with a prespecified reference category for each variable. Responses containing more than 20% missing data, duplicate submissions, and responses from participants who did not provide consent were excluded. The final analysis was based on complete eligible questionnaires, and no imputation was performed for missing questionnaire responses.

Content validity, pilot testing, and reliability assessment

The preliminary questionnaire underwent face- and content-validity assessments by a multidisciplinary expert panel. Each item was evaluated for relevance, clarity, simplicity, and appropriateness, and minor wording modifications were incorporated, based on expert recommendations, to improve readability and eliminate ambiguity. A pilot study involving 30 dental professionals representing different specialties was conducted to assess the comprehensibility, feasibility, and average time required to complete the questionnaire. Responses obtained during the pilot phase were not included in the final analysis. Feedback from pilot participants resulted in minor modifications to the sentence structure and wording without altering the conceptual framework of the questionnaire.

The internal consistency reliability of the final 12-item readiness scale was assessed using the Cronbach's alpha coefficient. Item-total statistics, corrected item-total correlations, and Cronbach's alpha if items were deleted were calculated to determine the contribution of individual items to the overall construct. The 12 readiness items were designed to capture complementary facets of AI readiness, including knowledge, perceived usefulness, confidence, trust, willingness to adopt AI, educational needs, and concerns regarding professional displacement. These domains were considered interrelated components of the broader concept of readiness for AI-assisted CBCT interpretation. Accordingly, the items were combined to generate an overall composite readiness score, with higher scores representing greater overall readiness. The composite score should be interpreted as a multidimensional indicator of readiness rather than as evidence that all items represent a single homogeneous latent dimension.

Questionnaire distribution and data collection

The finalized questionnaire was converted into a secure Google Forms (Google LLC, Mountain View, California, United States) survey and distributed electronically through professional dental networks, institutional mailing lists, and social media platforms including WhatsApp (Meta Platforms, Inc., Menlo Park, California, United States), Telegram (Telegram FZ-LLC, Dubai, United Arab Emirates), and email. The survey link remained active throughout the study. To maximize participation, reminder messages were circulated at two-week intervals. Google Forms was configured to restrict multiple submissions from the same Google account, and duplicate responses identified during data screening were removed before the analysis. Participant anonymity was maintained throughout the study, and no personally identifiable information was collected.

Outcome measures

The primary outcome measure was the overall AI readiness score, obtained by summing the responses to the 12 readiness items. Secondary outcome measures included readiness according to age, sex, educational qualification, years of clinical experience, and practice setting, together with the identification of independent predictors influencing AI readiness for CBCT interpretation.

Statistical analysis

Data were exported from Google Forms to Microsoft Excel (Microsoft Corporation, Redmond, Washington, United States) and analyzed using IBM SPSS Statistics for Windows, version 25.0 (IBM Corp., Armonk, New York, United States). Continuous variables were expressed as mean ± standard deviation (SD), whereas categorical variables were summarized as frequencies and percentages. The internal consistency of the questionnaire was evaluated using Cronbach's alpha, corrected item-total correlations, and Cronbach's alpha if an item was deleted. Independent-sample t-tests were used for subgroup comparisons with effect sizes reported as Cohen's d. Spearman's rank-order correlation was performed to evaluate the associations between demographic variables and readiness scores. Multiple linear regression analysis was conducted to identify independent predictors of AI readiness after assessing multicollinearity using variance inflation factors (VIF). Statistical significance was established at p < 0.05 using two-tailed tests.

## Results

A total of 380 dental professionals participated in the study. The largest age group was 31-40 years, followed by 20-30 years and 41-50 years. The majority of participants were male (n=209, 55.0%), while there were 171 female participants (45.0%). Most participants had an MDS qualification. The largest experience category was 5-10 years. Private group practice was the most common setting. AI readiness scores decreased with increasing age and clinical experience. PhD-qualified participants had the highest readiness score (33.8 ± 8.1), while participants with prior AI exposure scored higher (32.5 ± 7.4) than those without exposure (28.4 ± 8.2) (Table 1).

**Table 1:** Demographic characteristics and total AI readiness scores of the study participants (N = 380) SD: standard deviation; AI: artificial intelligence; BDS: Bachelor of Dental Surgery; MDS: Master of Dental Surgery

Demographic variable	Category	Frequency (Percentage)	AI Readiness score, mean ± SD
Age group	20–30 years	106 (27.9)	33.1 ± 8.2
	31–40 years	133 (35.0)	31.8 ± 8.9
	41–50 years	84 (22.1)	28.7 ± 9.5
	51–60 years	45 (11.8)	26.1 ± 10.1
	>60 years	12 (3.2)	22.4 ± 11.2
Sex	Male	209 (55.0)	31.5 ± 8.7
	Female	171 (45.0)	29.8 ± 9.4
Qualification	BDS	68 (17.9)	27.4 ± 9.7
	Fellowship/Diploma	46 (12.1)	30.5 ± 9.0
	MDS	236 (62.1)	31.2 ± 8.8
	PhD	30 (7.9)	33.8 ± 8.1
Clinical experience	<5 years	84 (22.1)	32.7 ± 8.3
	5–10 years	114 (30.0)	31.9 ± 8.7
	11–15 years	95 (25.0)	29.5 ± 9.1
	16–20 years	57 (15.0)	28.0 ± 9.6
	>20 years	30 (7.9)	25.8 ± 10.4
Practice setting	Private solo	76 (20.0)	28.5 ± 9.5
	Private group	114 (30.0)	30.1 ± 9.0
	Academic institution	95 (25.0)	33.2 ± 8.4
	Hospital/Government	57 (15.0)	30.8 ± 9.2
	Mixed (Academic + Clinical)	38 (10.0)	31.6 ± 8.8
Prior AI exposure	Yes	225 (58.9)	32.5 ± 7.4
	No	156 (41.1)	28.4 ± 8.2

**Figure 1:**
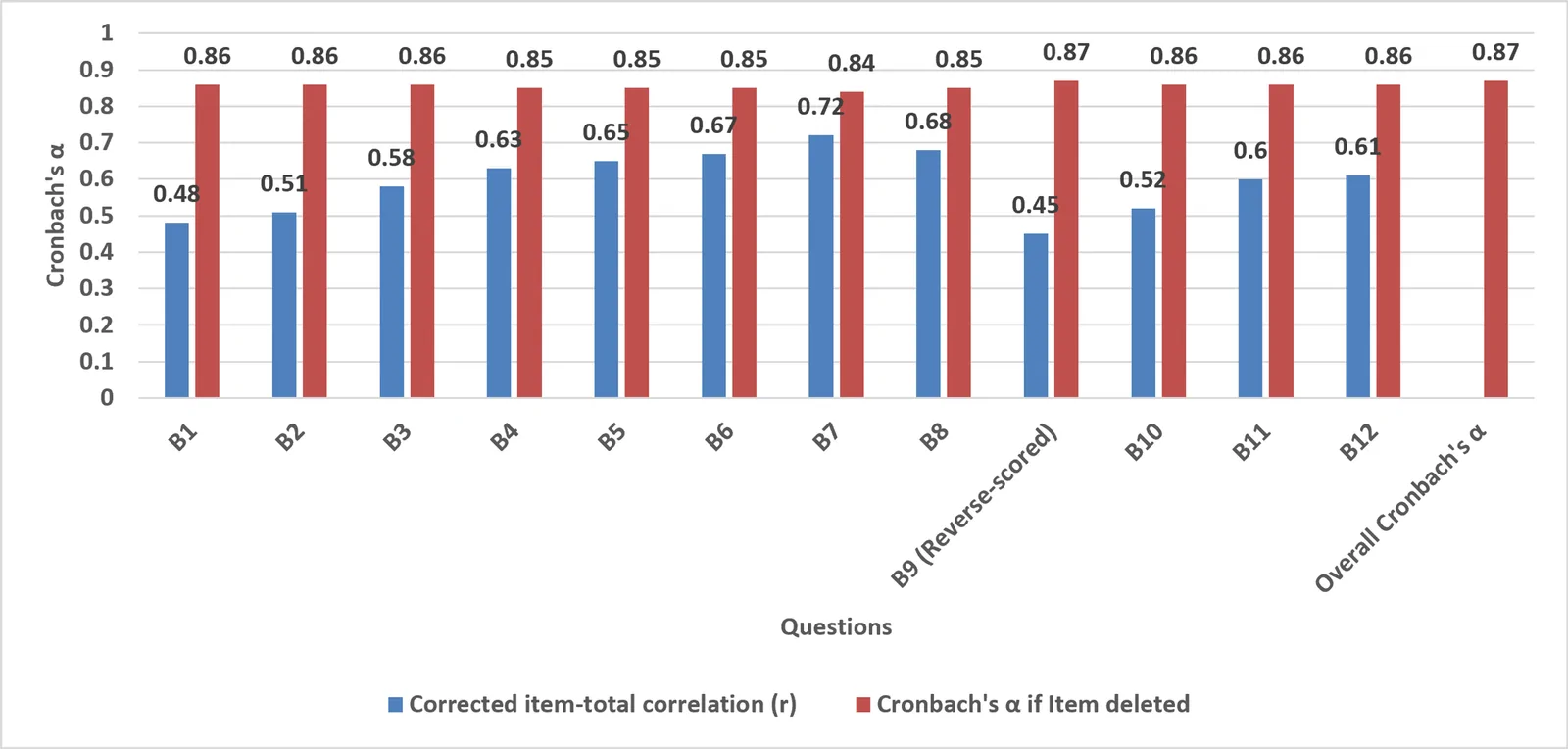
Internal consistency and item-total statistics of the 12-item AI readiness questionnaire Data are presented as corrected item-total correlation (r) and Cronbach's alpha if item deleted, internal consistency was assessed using Cronbach's alpha reliability analysis, B9 was reverse-scored before reliability analysis B: questionnaire item; r: corrected item-total correlation; α: Cronbach's alpha

The psychometric evaluation of the 12-item AI readiness questionnaire demonstrated good internal consistency, with an overall Cronbach's alpha of 0.87. The corrected item-total correlations ranged from 0.45 to 0.72, with the highest value observed for B7 (0.72) and the lowest for the reverse-scored B9 (0.45). Cronbach's alpha if an item was deleted ranged from 0.84 to 0.87, indicating that the deletion of any individual question did not improve the overall reliability of the instrument. These findings confirm that all questionnaire items contributed meaningfully to the assessment of AI readiness (Figure 1).

The significant differences in AI readiness scores were observed across all the evaluated demographic variables. Male participants demonstrated significantly higher readiness scores than did female participants (t = 2.04, p = 0.042, Cohen's d = 0.21). Participants aged ≤40 years exhibited significantly greater readiness than those aged >40 years (t = 3.89, p < 0.001, d = 0.40). Similarly, postgraduate qualified professionals (MDS/PhD) showed significantly higher readiness than BDS graduates and fellowship or diploma holders (t = 4.12, p < 0.001, d = 0.44). Dental professionals with <10 years of clinical experience demonstrated greater readiness than those with ≥10 years of experience (t = 4.01, p < 0.001, d = 0.42). Academic practitioners also exhibited significantly higher readiness than non-academic practitioners (t = 3.56, p < 0.001, d = 0.37), with all significant comparisons demonstrating small to moderate effect sizes (Table 2).

**Table 2:** Comparison of total AI readiness scores across demographic subgroups Data are presented as independent sample t-test statistics with effect size (Cohen's d) and 95% confidence intervals, df: degree of freedom, comparisons were performed using the independent sample t-test *p < 0.05 was considered statistically significant AI: artificial intelligence

Independent variable	Comparison groups	Test statistic	df	p-value	Effect size	95% CI for Effect size
Sex	Male vs. Female	2.04	378	0.042*	0.21	0.01, 0.41
Age	≤40 years vs. >40 years	3.89	378	< 0.001*	0.40	0.20, 0.60
Qualification	Postgraduate (MDS/PhD) vs. Graduate (BDS/Fellowship)	4.12	378	< 0.001*	0.44	0.23, 0.65
Experience	<10 years vs. ≥10 years	4.01	378	< 0.001*	0.42	0.21, 0.62
Practice type	Academic vs. Non-Academic (Private/Hospital)	3.56	378	< 0.001*	0.37	0.17, 0.57

Age was strongly positively correlated with clinical experience (r = 0.86, p < 0.001). Both age (r = −0.34, p < 0.001) and clinical experience (r = −0.32, p < 0.001) were negatively associated with overall AI readiness scores, whereas qualification level showed a modest positive correlation with readiness (r = 0.28, p < 0.001). Qualification was weakly negatively correlated with age (r = −0.24) and clinical experience (r = −0.20) (Table 3).

**Table 3:** Spearman's rank-order correlation between demographic characteristics and AI readiness scores Data are presented as Spearman's rank-order correlation coefficients (r), correlations were analyzed using Spearman's rank-order correlation test *indicates statistical significance at p < 0.05 AI: artificial intelligence

Variable	Age	Experience	Qualification	Total score
Age	1.00	0.86*	-0.24*	-0.34*
Clinical experience	0.86*	1.00	-0.20*	-0.32*
Qualification	-0.24*	-0.20*	1.00	0.28*
Total readiness score	-0.34*	-0.32*	0.28*	1.00

The regression model was statistically significant (F = 25.11, p < 0.001) and explained 25% of the variance in AI readiness (R² = 0.25, adjusted R² = 0.24). Greater clinical experience independently predicted lower readiness (β = −0.28, p < 0.001), whereas higher qualification level (β = 0.15, p = 0.002), academic practice setting (β = 0.10, p = 0.042), and prior AI training (β = 0.17, p = 0.001) were significant positive predictors. Age was not an independent predictor after adjusting for the other variables (β = −0.09, p = 0.231). Variance inflation factor values ranged from 1.08 to 4.02, indicating no significant multicollinearity, while the Durbin-Watson statistic of 1.94 confirmed the independence of residuals (Table 4).

**Table 4:** Multiple linear regression analysis of predictors of AI readiness for CBCT interpretation Model summary: R = 0.50, R^2^ = 0.25, adjusted R^2^ = 0.24, F (5,374) = 25.11, p < 0.001, Durbin-Watson = 1.94. Multiple linear regression analysis was performed to identify independent predictors of AI readiness *p < 0.05 was considered statistically significant β: standardized regression coefficient; B: unstandardized regression coefficient; VIF: variance inflation factor; R²: coefficient of determination; AI: artificial intelligence; CBCT: cone-beam computed tomography

Predictor variable	Unstandardized β (B)	Standard error	Standardized β (Beta)	t-value	p-value	VIF
(Constant)	18.44	2.51	-	7.35	< 0.001*	-
Clinical experience (years)	-0.85	0.21	-0.28	-4.05	< 0.001*	3.91
Qualification level	1.21	0.39	0.15	3.10	0.002*	1.12
Practice setting (Academic vs. Others)	0.55	0.27	0.10	2.04	0.042*	1.08
Prior AI training (Yes/No)	1.94	0.56	0.17	3.46	0.001*	1.12
Age (years)	-0.18	0.15	-0.09	-1.20	0.231	4.02

## Discussion

The present cross-sectional survey evaluated the readiness of dental professionals to adopt AI for CBCT interpretation and identified the demographic and professional factors influencing its acceptance. Overall, the findings demonstrated a favorable level of readiness among dental professionals, although acceptance varied significantly according to age, educational qualifications, clinical experience, and practice setting. These findings suggest that, while AI is increasingly recognized as a valuable adjunct for CBCT interpretation, its successful implementation depends on improving awareness, education, and structured training among practicing clinicians.

Demographic analysis revealed that younger dental professionals and those with fewer years of clinical experience exhibited significantly greater AI readiness than older and more experienced practitioners. This observation is consistent with previous studies, which reported early career clinicians were generally more receptive to digital technologies because they had greater exposure to AI, digital imaging, and computer-assisted diagnostics during undergraduate and postgraduate education [8,11,12]. Conversely, senior practitioners often rely on established diagnostic approaches and may demonstrate greater caution toward adopting emerging technologies because of concerns regarding reliability, workflow disruption, and medicolegal accountability. Similar observations were reported by Gupta et al., who found a weak negative correlation between AI knowledge, attitudes, and perceptions with increasing age and years of professional experience among orthodontists and postgraduate students [8]. Similarly, Hegde et al. reported that attitudes toward AI applications in dentistry were significantly influenced by age, clinical experience, and technological proficiency, with younger professionals demonstrating greater optimism toward AI adoption [13].

Educational qualifications also influenced readiness, with postgraduate and PhD-qualified professionals demonstrating significantly higher AI readiness than BDS graduates. Higher academic qualifications are generally associated with greater involvement in research, evidence-based practice, and continuing professional development, which increases awareness of recent technological advancements. Specialists frequently use CBCT for complex diagnosis and treatment planning in implant dentistry, orthodontics, oral and maxillofacial surgery, and endodontics, making them more familiar with AI-assisted image analysis [14]. This finding is consistent with recent evidence indicating that higher levels of education and professional training are associated with greater AI literacy, stronger confidence in AI-assisted clinical applications, and greater willingness to integrate AI into routine dental practice [15]. Furthermore, international experts have emphasized that structured AI education at both undergraduate and postgraduate levels is essential for developing the competencies required for the safe and effective implementation of AI in dentistry [16].

Practical settings have emerged as another significant determinant of AI readiness. Participants working in academic institutions demonstrated higher readiness scores than those working exclusively in private or hospital settings. Academic clinicians are routinely exposed to research activities, technological innovations, continuing education programs, and collaborative interdisciplinary discussions, all of which facilitate the early adoption of novel technologies [17,18]. Universities also provide greater access to advanced imaging equipment, AI software demonstrations, and educational workshops, encouraging positive attitudes toward AI-assisted diagnosis [19].

The present study demonstrated the excellent internal consistency of the AI readiness questionnaire, with a Cronbach's alpha of 0.87, confirming that the instrument reliably measured readiness for AI-assisted CBCT interpretation. Appropriate corrected item-total correlations and stable Cronbach's alpha values after the deletion of individual items indicated that each statement contributed meaningfully to the overall construct. The availability of a reliable and validated questionnaire provides an important tool for future surveys assessing AI adoption among dental professionals, and may facilitate comparisons across different populations and healthcare systems.

Correlation analysis demonstrated that increasing age and clinical experience were negatively associated with AI readiness, whereas higher educational qualifications were positively associated [20]. Interestingly, multiple regression analysis revealed that clinical experience remained an independent negative predictor, whereas higher qualifications, academic practice setting, and prior AI training independently increased readiness. Age lost statistical significance after adjustment, suggesting that differences attributed to age may largely reflect disparities in educational exposure and previous AI training, rather than chronological age itself. These findings emphasize that adequate education and structured training may overcome resistance to AI adoption irrespective of age [21].

The overall findings support the growing evidence that dental professionals perceive AI primarily as a decision support system rather than a replacement for clinical expertise. Participants expressed favorable attitudes toward integrating AI into CBCT interpretation, while simultaneously acknowledging the importance of adequate professional training before implementation. Similar conclusions have been drawn by recent systematic reviews, which indicate that clinicians are more willing to adopt AI when its recommendations remain transparent, explainable, and under the supervision of qualified dental professionals [22,23].

Although the overall readiness toward AI-assisted CBCT interpretation was favorable, alternative explanations should also be considered. Participants with greater interest in digital technologies or AI may have been more inclined to participate in an online survey, resulting in self-selection of individuals who were already positively disposed toward AI. Recruitment through professional networks and electronic platforms may similarly have preferentially reached digitally engaged dental professionals. In addition, favorable responses may partly reflect social desirability or the perception that AI represents an important future direction in dentistry. Importantly, perceived readiness does not necessarily translate into actual clinical adoption. Practical barriers, including access to appropriate AI systems, training requirements, cost, infrastructure, explainability, professional autonomy, and medico-legal responsibility, may influence whether favorable attitudes ultimately result in implementation. Therefore, the relatively high readiness scores observed in this study should be interpreted as an indicator of self-reported willingness and perceived preparedness rather than definitive evidence of actual clinical readiness for AI integration.

Clinical implications

The findings suggest that dental professionals demonstrate a generally favorable readiness to consider AI-assisted CBCT interpretation. This favorable readiness may facilitate future implementation of AI-based decision-support systems in dental practice when appropriate training, infrastructure, and professional oversight are available. However, the present study did not directly evaluate diagnostic accuracy, interpretation time, clinical efficiency, or patient outcomes. Therefore, any potential benefits of AI-assisted CBCT interpretation should be regarded as areas for further investigation rather than outcomes demonstrated by this study. Future clinical studies should evaluate whether AI-assisted interpretation can improve diagnostic performance, consistency, workflow efficiency, and patient care while maintaining appropriate professional oversight.

Limitations

The present study has several limitations. The newly developed questionnaire demonstrated good internal consistency and preliminary face and content validity; however, comprehensive psychometric validation was not performed. The electronic recruitment strategy may have introduced selection bias, as digitally engaged or AI-interested professionals may have been more likely to participate. Although recruitment was open across India, the study was coordinated from a single institution in Gujarat, which may limit geographical generalizability. In addition, self-reported responses may have been affected by social desirability bias. Finally, AI readiness was assessed at a single time point, and actual AI use, objective competency, and clinical performance were not evaluated; therefore, the findings reflect perceived readiness rather than actual clinical adoption or effectiveness. In addition, prior AI exposure, institutional resources, and access to training may have influenced readiness scores and may represent potential confounding factors. These considerations should be taken into account when interpreting the findings and their generalizability.

Future recommendations

Future multicenter studies involving larger and more diverse populations are recommended to improve the generalizability of our findings. Longitudinal studies should evaluate the changes in AI readiness following structured educational interventions and continuing professional development programs. Future research should also validate this questionnaire across different countries and healthcare settings, assess specialty-specific perceptions, and investigate the relationship between AI readiness and clinical adoption. Comparative studies evaluating different AI platforms for CBCT interpretation and their influence on diagnostic accuracy, clinical decision-making, and patient outcomes would further strengthen the evidence supporting the integration of AI into dental radiology.

## Conclusions

Dental professionals demonstrated generally favorable self-reported readiness to adopt AI for CBCT interpretation, with readiness varying according to educational qualification, clinical experience, practice setting, and prior AI training. The questionnaire demonstrated good internal consistency and preliminary evidence of face and content validity. These findings suggest an encouraging level of acceptance toward AI-assisted CBCT interpretation; however, they do not establish its effectiveness, diagnostic accuracy, or clinical efficiency. Structured AI education and professional training may support responsible future adoption, while the actual impact of AI-assisted CBCT interpretation on diagnostic performance and clinical outcomes should be evaluated through appropriately designed clinical studies.

## Appendices

**Table 5:** Study questionnaire

Section !: Demographic details		
Q.No.	Question	Response Options
A1	What is your age group?	□ 20–30 years
		□ 31–40 years
		□ 41–50 years
		□ 51–60 years
		□ >60 years
A2	What is your gender?	□ Male
		□ Female
A3	What is your highest professional qualification?	□ BDS
		□ MDS (specify specialty: _____)
		□ Fellowship/Diploma
		□ PhD
		□ Other (please specify: _____)
A4	How many years of clinical experience do you have?	□ <5 years
		□ 5–10 years
		□ 11–15 years
		□ 16–20 years
		□ >20 years
A5	What is your primary practice setting?	□ Private practice (solo)
		□ Private practice (group/corporate)
		□ Academic institution
		□ Hospital/Government institution
		□ Mixed (academic + clinical)
		□ Other (please specify: _____)
A6	Have you received any formal training in artificial intelligence (AI) or AI-assisted dental applications?	□ Yes
		□ No
Section 2: AI Readiness		
Q.No.	Statement	Score given
B1	I am familiar with the basic concepts of artificial intelligence and its applications in dentistry.	
B2	I have adequate knowledge about the potential role of AI in interpreting CBCT images.	
B3	I believe AI can assist in the accurate detection of pathological lesions on CBCT scans.	
B4	I trust AI-generated interpretations of CBCT images when used as a supplementary diagnostic tool.	
B5	I would feel confident relying on AI assistance for differentiating vital and pathological structures on CBCT.	
B6	I believe that AI can improve the accuracy and consistency of CBCT interpretation in dental practice.	
B7	I am willing to incorporate AI-based CBCT interpretation software into my clinical workflow.	
B8	I believe that AI can enhance treatment planning decisions when integrated with CBCT analysis.	
B9	I am concerned that AI may reduce the role of dental professionals in CBCT interpretation.	
B10	I believe that adequate training and education are necessary before adopting AI for CBCT interpretation.	
B11	I would recommend the use of AI-assisted CBCT interpretation to my colleagues or patients.	
B12	I believe AI has the potential to significantly improve early detection and diagnosis of oral pathologies through CBCT analysis.	
Scoring- 0: strongly disagree, 1: Disagree, 2: Neutral, 3: Agree, 4: Strongly agree.		
Interpretation: maximum score 48 and minimum score 0		
